# Postmarketing Safety Monitoring After Influenza Vaccination Using a Mobile Health App: Prospective Longitudinal Feasibility Study

**DOI:** 10.2196/26289

**Published:** 2021-05-07

**Authors:** Minh Tam H Nguyen, Gérard Krause, Brigitte Keller-Stanislawski, Stephan Glöckner, Dirk Mentzer, Jördis J Ott

**Affiliations:** 1 PhD Programme Epidemiology Hannover Biomedical Research School Hannover Medical School Hannover Germany; 2 Hannover Medical School Hannover Germany; 3 Paul Ehrlich Institute Langen Germany

**Keywords:** mHealth, mobile health, digital health, adverse event, adverse event following immunization, active reporting, pharmacovigilance, therapeutic use, adverse effect

## Abstract

**Background:**

For the safety monitoring of vaccinations postlicensure, reports of adverse events after immunization (AEFIs) are crucial. New technologies such as digital mobile apps can be used as an active approach to capture these events. We therefore conducted a feasibility study among recipients of the influenza vaccination using an app for assessment of the reporting of AEFIs.

**Objective:**

The goal of the research was to determine factors influencing adherence to and correct use of a newly developed app for individuals to report AEFI for 3 months using regular reminder functions, to identify determinants of AEFI occurrence and define reported AEFI types.

**Methods:**

We developed the app (SafeVac) and offered it to recipients of the influenza vaccination in 3 occupational settings in fall 2018. In this prospective longitudinal feasibility study, data on AEFIs were generated through SafeVac for 3 months. Using logistic and Cox regression, we assessed associations between app adherence, correct app entry, AEFIs, and sociodemographic parameters.

**Results:**

Of the individuals who logged into SafeVac, 61.4% (207/337) used the app throughout a 3-month period. App use adherence was negatively associated with female sex (odds ratio [OR] 0.47; CI 0.25-0.91) and correct app entry was negatively associated with older age (OR 0.96; CI 0.93-0.99) and lower education (OR 0.31; CI 0.13-0.76). AEFI occurrence was associated with female sex (hazard ratio 1.41; CI 1.01-1.96) and negatively with older age (hazard ratio 0.98; CI 0.97-0.99). The most common AEFIs reported were injection site pain (106/337), pain in extremity (103/337), and fatigue/asthenia (73/337).

**Conclusions:**

Digital AEFI reporting was feasible with SafeVac and generated plausible results for this observation period and setting. Studies directly comparing SafeVac with conventional passive reporting schemes could determine whether such digital approaches improve completeness, timeliness, and sensitivity of vaccine vigilance. Further studies should evaluate if these results are transferable to other vaccinations and populations and if introduction of such a tool has an influence on vaccination readiness and therefore vaccine safety.

## Introduction

Vaccinations have been the most effective measure to prevent infectious diseases, preventing over 2 million deaths per year worldwide [1,2]. Especially for new and changing vaccines (eg, those against COVID-19 or the seasonal flu), intensified and timely surveillance of adverse events following immunization (AEFIs) might be of high relevance [3,4]. This is also indicated because these vaccinations are administered to many individuals with different characteristics and medical histories within a short period of time [4-6]. Although all licensed vaccines undergo clinical safety trials until they obtain regulatory approval, some AEFI become known only after marketing on a large scale [7,8]. In Germany, physicians are obliged by law to report suspicions of health impairments that exceed the normal degree of a vaccination to the local health authority (§6 Abs. 1, no. 3, IfSG), which is obliged to report to the Paul Ehrlich Institute (PEI), the Federal Institute for Vaccines and Biomedicines, via phone, mail, fax, or online. Vaccination recipients can also voluntarily contribute to vaccine safety via a web-based app [9]. While this spontaneous reporting system is a cost-effective way to detect adverse events, it is limited by imprecise information on the denominator, underreporting, and delay in reporting due to various media breaches in reporting [10-12]. A new way to capture AEFIs and overcome these limitations is to have patients send reports directly using a mobile app. Although more than 318,000 health apps were available in the app stores in 2017, the apps were primarily developed for exercises and fitness [13] rather than to capture adverse events. In fact, in a systematic review by Cashman et al [14], only a single participant-centered app capturing AEFIs in near real time was found [15]. This clearly shows a general lack of research on an app to report AEFIs, especially in long-term use. The long period of monitoring is particularly important to capture previously unknown unexpected AEFIs with late onset. Furthermore, in epidemiological studies, longitudinal data can help to assess causal relationships and explore determinants [16].

Therefore, our aim was to (1) assess whether an app for reporting AEFIs can be used for 3 months, (2) determine factors influencing the adherence to and correct use of a newly developed app (SafeVac, Paul Ehrlich Institute, and Helmholtz Centre for Infection Research [HZI]) for individuals to report AEFIs for 3 months using regular reminder functions, and (3) identify determinants for AEFI occurrence and define reported AEFI types.

## Methods

### Participant Recruitment

We recruited participants for our prospective longitudinal study from staff of 3 different employers in Germany during fall 2018: Investitionsbank Berlin, PEI, and University Hospital Frankfurt. We included volunteers on 2 respective days. To be eligible, individuals had to be employed at these institutions, vaccinated against influenza by the occupational health physician on these days, own a smartphone, be at least 18 years old, and be proficient in the German language.

We gave participants who agreed to take part in our study an information leaflet comprising information about the study, data transmission, and data storage; a random ID; and instructions on downloading and using the SafeVac app. Once the individuals agreed to participate and signed the declaration of consent, we asked participants to download and log in to SafeVac immediately after receiving the influenza vaccination. In the app, participants provided sociodemographic information and their vaccination history and then entered information about occurrence or nonoccurrence of adverse events. After participants used the app, we distributed usability questionnaires based on the System Usability Scale (SUS) and evaluated them using the adjective rating scale [17,18]. Additionally, participants could take part in a lottery to win a tablet or smartwatch. This was offered to all participants who completed the AEFI reports for at least 1 week.

The study protocol and data protection concept were reviewed and assessed without any concerns by the ethical committee of the Medical Association of Lower Saxony in Germany, the HZI institutional data protection officer, and the Federal Commissioner for Data Protection and Freedom of Information.

### AEFI Reporting and Conception of the SafeVac App

In SafeVac, we asked participants to enter information on receipt of influenza and previous vaccinations and sociodemographic variables and describe any AEFI occurrence. We asked these questions at 15 intervals defined over a period of 3 months: 1 hour, 4 hours, and 8 hours after vaccination; daily until the 7th day after vaccination; weekly for 3 weeks after vaccination; and monthly for 2 subsequent months after vaccination. In case of AEFI occurrence, participants answered additional questions in the app based on requirements of the online reporting platform for adverse events hosted by the national responsible authorities PEI and the Federal Institute for Drugs and Medical Devices. Participants could select a specific AEFI (eg, fatigue) from a drop-down list or enter it manually in the comment text field. In addition, participants were asked to provide additional information about chronic medical conditions, medications taken for chronic conditions, and pregnancy status.

**Figure 1:**
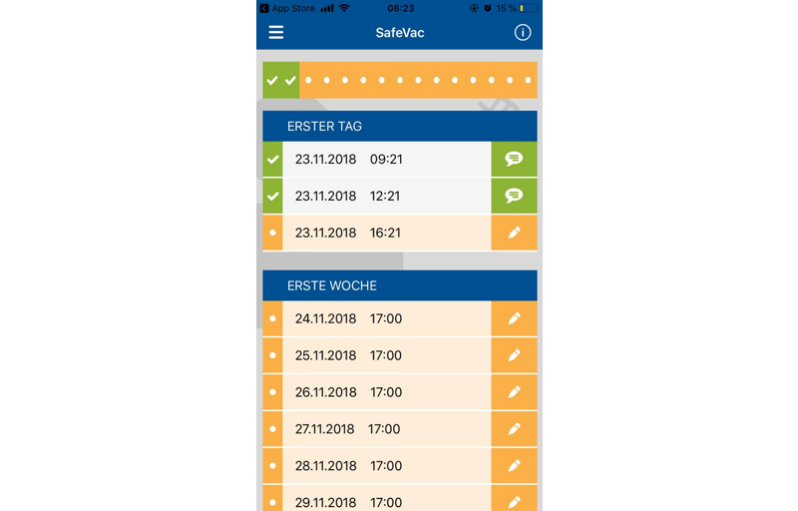
Screenshot of SafeVac app.

SafeVac was available in Android (version 4.4 or higher) and iOS (10 or higher) app stores. For the development and design of SafeVac, we took a user-centered approach for which we gathered data in a previous study on users’ app preferences [19]. Based on these results, we used gamification elements such as the appearance of puzzle pieces and a loading bar to enhance app use adherence (Figure 1). Every app entry was transmitted anonymously and securely to PEI using https/SSL encryption. To evaluate the feasibility of the app, we used the adherence rate as a surrogate parameter and assessed whether AEFIs entered into the app were in accordance with the literature.

### Data Management and Data Analysis

We included all information collected through SafeVac from fall 2018 until March 14, 2019, in our study. Before analyzing the data, we excluded test IDs. We coded AEFI entries in the free-text fields according to the preferred terms in the Medical Dictionary of Regulatory Activities and validated them using the four-eyes principle. We also checked for consistency in cases where participants had entered AEFIs manually in the comment text field and additionally selected them from the drop-down list at the same time point. In case of discrepancy, we coded them as 2 different types of AEFIs. If participants entered 2 identical AEFIs for the same time point, we removed one. If the free-text field described the AEFI in more detail than the AEFI selected on the drop-down list, we ignored the selected AEFI and coded the manually entered AEFI systematically according to the corresponding preferred term (eg, if “pain in extremities” was selected in the drop-down list and “pain in the arm (injection site)” was entered manually, we coded the AEFI as “injection site pain”). The BMI was calculated and categorized using the World Health Organization scale approach [20].

By using logistic regression, we estimated the relationship between sociodemographic variables and vaccination uptakes with the binary outcomes app adherence until the end of the study, AEFI occurrence and correct entry of vaccination information, respectively. We defined incorrect entry of vaccination information as any misspellings in the name of the received influenza vaccination or its associated batch number but did not take case sensitivity into account. To find determinants for the outcome AEFI occurrence in 3 months, we used a Cox regression model. Variables were selected by using a backward selection by Akaike information criterion. In all models we set age and sex as a priori confounders. Missing data were not included in the model.

Additionally, we used Cox regression to explore determinants of reporting an influenza-like illness as an AEFI. We used R software version 3.2.5 (R Foundation for Statistical Computing) for data analysis and visualization.

### Study Participation and Evaluation of SafeVac

To assess quality and usability of the app, we developed a feedback questionnaire in LimeSurvey, an online survey tool, by including the SUS and using an approach of the User Version of the Mobile Application Rating Scale [21,22]. We distributed the feedback questionnaire through a link 1 year after the start of the study via the internal websites of the 3 participating institutions.

## Results

### Participant Characteristics and AEFI Reporting Over Three Months

**Figure 2:**
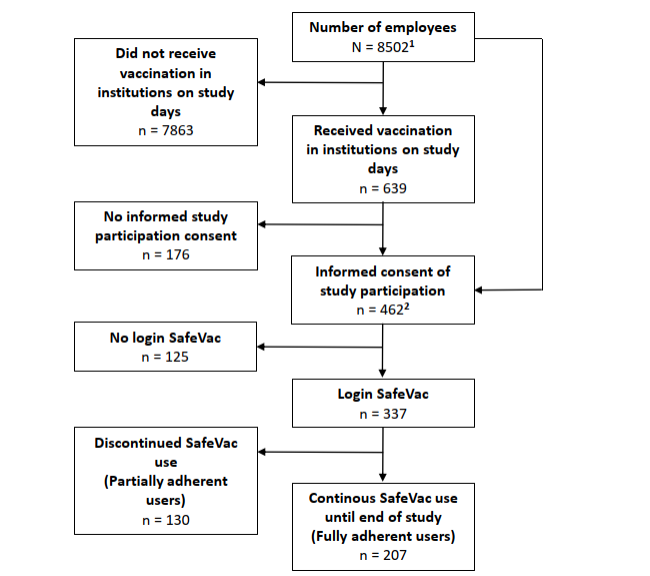
Study participant recruitment. ^1^Obtained through personal communication and estimates based on institutional website; for PEI and IBB only ≥18 years old included; ^2^included participants who were vaccinated before study days.

Of the participants who provided informed consent, 72.9% (337/462) logged into the SafeVac (study population) and made a minimum of one app entry; 61.4% (207/337) used the app until the end of the study (Figure 2).

The majority of participants were female (224/337, 66.5%) and 84.0% (283/337) had a general certificate of education (Abitur). Almost half of the participants (166/337, 49.3%) stated that they had also been vaccinated against influenza in an earlier year. We assessed characteristics according to adherence of app use in the study (fully vs partially adherent participants), and they appear mostly similar (eg, 74/207 [35.7%] vs 39/130 [30.0%] male participants; Table 1).

**Table 1:** Characteristics of study participants according to adherence to app use.

Characteristic			Fully adherent participants^a^ (n=207)		Partially adherent participants^b^ (n=130)		Total (n=337)
Age in years, mean			35.7		37.3		36.3
**Sex, n (%)**							
	Male	74 (35.7)		39 (30.0)		113 (33.5)	
	Female	133 (64.3)		91 (70.0)		224 (66.5)	
**General certificate of education, n (%)**							
	Yes	172 (83.1)		111 (85.4)		283 (84.0)	
	No	31 (15.0)		17 (13.1)		48 (14.2)	
	Missing	4 (1.9)		2 (1.5)		6 (1.8)	
**Vaccination place, n (%)**							
	FFM^c^ (Frankfurt Main)	150 (72.5)		75 (57.7)		225 (66,8)	
	PEI^d^ (Langen)	23 (11.1)		22 (16.9)		45 (13.4)	
	IBB^e^ (Berlin)	30 (14.5)		30 (23.1)		60 (17.8)	
	Missing	4 (1.9 )		3 (2.3)		7 (2.1)	
**Vaccinated against influenza last year, n (%)**							
	Yes	103 (49.8)		59 (45.4)		162 (48.1)	
	No	28 (13.5)		24 (18.5)		52 (15.4)	
	Missing	76 (36.7)		47 (36.2)		123 (36.5)	
**Number of influenza vaccinations within the last 5 years, n (%)**							
	0	58 (28.0)		37 (28.5)		95 (28.2)	
	1	38 (18.4)		13 (10.0)		51 (15.1)	
	2	23 (11.1)		15 (11.5)		38 (11.3)	
	3	24 (11.6x)		19 (14.6)		43 (12.8)	
	4	21 (10.1)		16 (12.3)		37 (11.0)	
	5	37 (17.9)		24 (18.5)		61 (18.1)	
	Cannot remember	6 (29.0)		5 (3.8)		11 (3.3)	
	Missing	0		1 (0.8)		1 (0.3)	

^a^Fully adherent participants defined as participants who replied to questions on adverse event following immunization at all app notification time points.

^b^Partially adherent participants defined as participants who replied to questions on adverse event following immunization to some app notification time points.

^c^FFM: University Hospital Frankfurt*.*

^d^PEI: Paul Ehrlich Institute.

^e^IBB: Investitionsbank Berlin*.*

**Figure 3:**
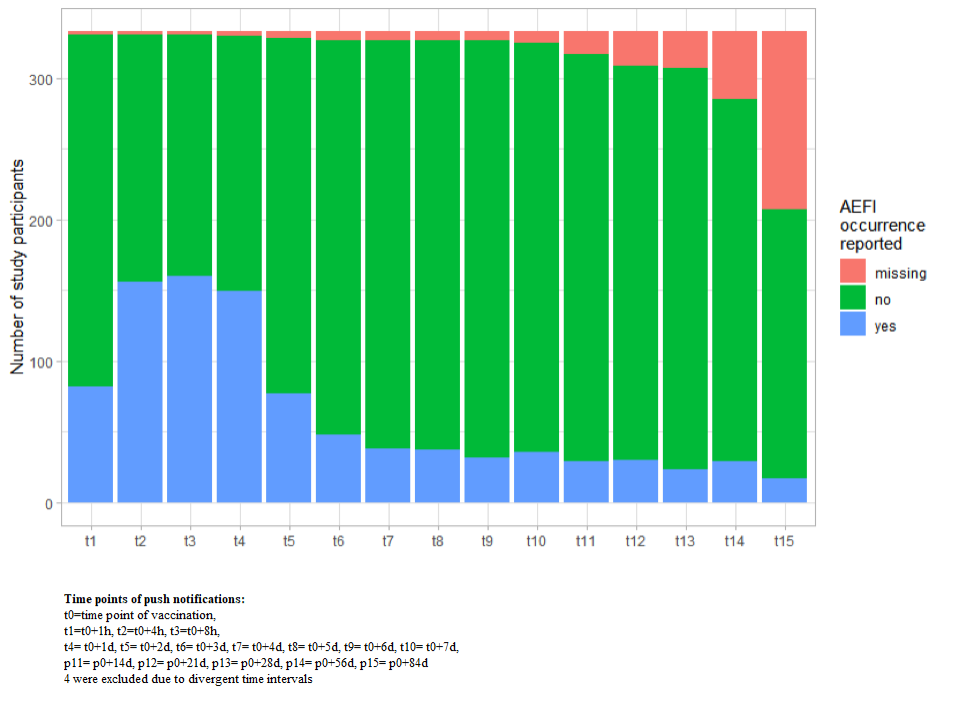
Reported adverse event following immunization occurrences per time point after influenza vaccination.

Of the participants who logged in, 81.4% (271/333) reported experiencing one or more AEFIs after vaccination. We found a rise in reported AEFIs at 4 hours after vaccination (156/333, 47.8%); thereafter, the number of participants reporting an AEFI remained steady over 8 hours (160/333, 48.0%) and 1 day (149/333, 44.7%) after vaccination. A decline started from 2 days after vaccination (77/333, 23.1%) onward. Study participant reporting attrition increased slightly over time, with the highest attrition difference (78) between 56 days and 84 days after vaccination (Figure 3).

### Factors Associated With Adherence to App Use

**Figure 4:**
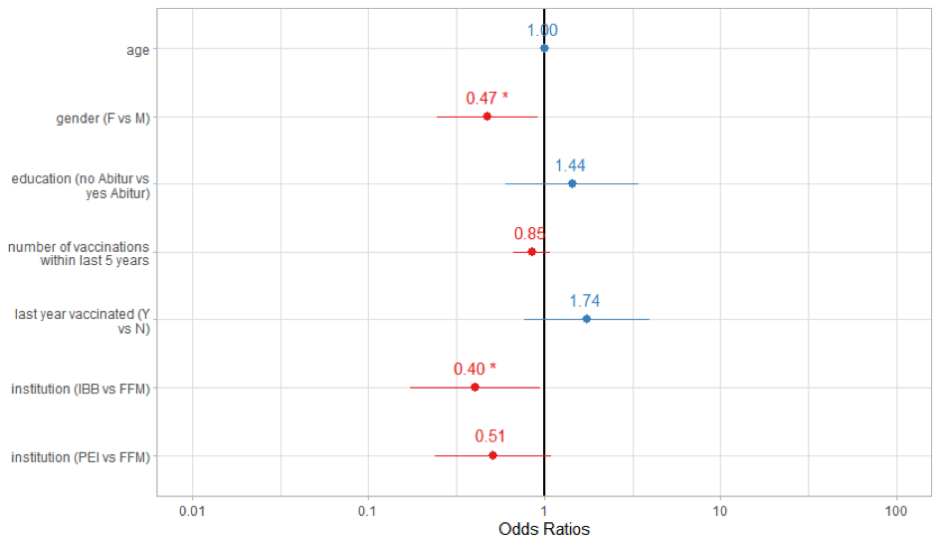
Factors associated with fully adherent app use versus partially adherent app use over 3 months, pseudoR2 (McFadden) = 0.065. F: female; M; male; Y: yes; N: no; IBB: Investitionsbank Berlin; FFM: University Hospital Frankfurt; PEI: Paul Ehrlich Institute.

Regarding adherence to SafeVac use until the end of follow-up, our logistic regression revealed a decrease of 43% (odds ratio [OR] 0.47; CI 0.25-0.91) for females and staff of the banking institution. The latter population was significantly less adherent than staff of the university hospital (OR 0.40; CI 0.17-0.94) after adjusting for age, sex, education, influenza vaccination received in the last year, number of influenza vaccinations in previous 5 years, occurrences of AEFIs, and institutional affiliation (Figure 4).

### Factors Associated With Correct App Entry of Vaccination

**Figure 5:**
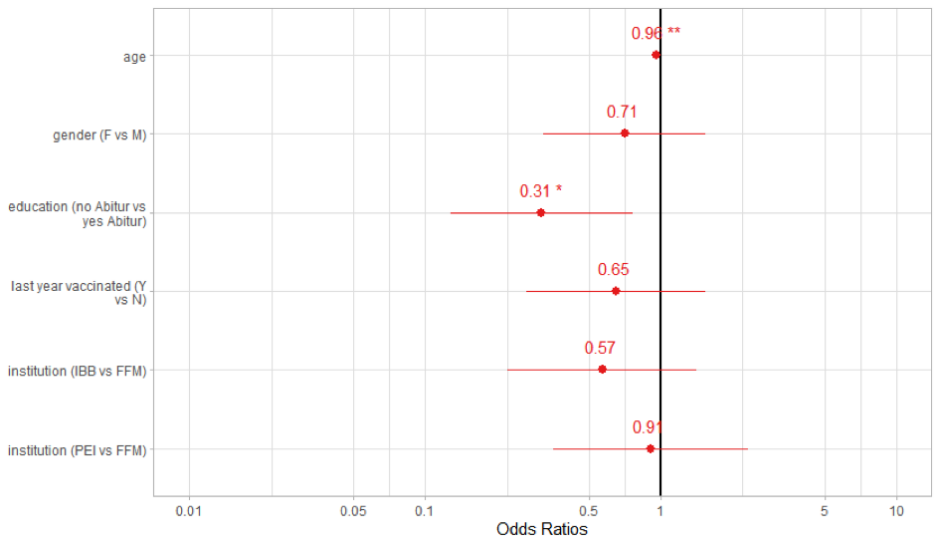
Factors associated with correct versus incorrect entry of vaccination information, pseudoR2 (McFadden) = 0.124. F: female; M; male; Y: yes; N: no; IBB: Investitionsbank Berlin; FFM: University Hospital Frankfurt; PEI: Paul Ehrlich Institute.

Overall, 260 participants correctly entered the data for their received vaccination. Having no Abitur (OR 0.31; CI 0.13-0.76) and increasing age (OR 0.96; CI 0.93-0.99) were negatively associated with correctness of entry of vaccine information (Figure 5). Other factors we analyzed (eg, age, gender, vaccination received last year, and affiliated institution) were not significantly associated with correct app entry of vaccination.

### Most Reported AEFI Occurrences From Study Participants

The most mentioned AEFIs from all participants were injection site pain (106/337, 31.5%), followed by pain in extremity (103/337, 30.6%) and fatigue/asthenia (73/337, 21.7%). Proportional differences in reporting were found in fully adherent and partially adherent participants (eg, pain in extremity, 60/207 [29.0%], 43/130 [33.1%]). However, there were no statistically significant differences in AEFI reporting between fully adherent and partially adherent app users (Table 2).

**Table 2:** Most common AEFIs according to app use adherence.

Adverse event	Fully adherent participants reporting AEFI^a^ (n=207), n (%)	Partially adherent participants reporting AEFI (n=130), n (%)	*P* value^b^	Participants reporting AEFI (n=337), n (%)
Injection site pain	66 (31.9)	40 (30.8)	.83	106 (31.5)
Pain in extremity	60 (29.0)	43 (33.1)	.42	103 (30.6)
Fatigue/asthenia	42 (20.3)	31 (23.8)	.44	73 (21.7)
Headache	39 (18.8)	23 (17.7)	.79	62 (18.4)
Influenza-like illness	38 (18.4)	18 (13.8)	.27	56 (16.6)
Myalgia	39 (18.8)	17 (13.1)	.17	56 (16.6)
Rhinitis	31 (15.0)	20 (15.4)	.91	51 (15.1)
Throat irritation	25 (12.1)	21 (16.2)	.29	46 (13.6)
Cough	27 (13.0)	12 (9.2)	.29	39 (11.6)
Malaise	12 (5.8)	6 (4.6)	.64	18 (5.3)
Local reaction	9 (4.3)	6 (4.6)	.90	15 (4.5)
Dizziness	9 (4.3)	3 (2.3)	.38	12 (3.6)
Mobility decreased	8 (3.9)	3 (2.3)	.54	11 (3.3)
Injection site swelling	5 (2.4)	5 (3.8)	.52	10 (3.0)

^a^AEFI: adverse event following immunization.

^b^Pearson chi-square test for cells n>5 and Fisher exact test for cells n≤5.

### Factors Associated With AEFI Occurrence

**Figure 6:**
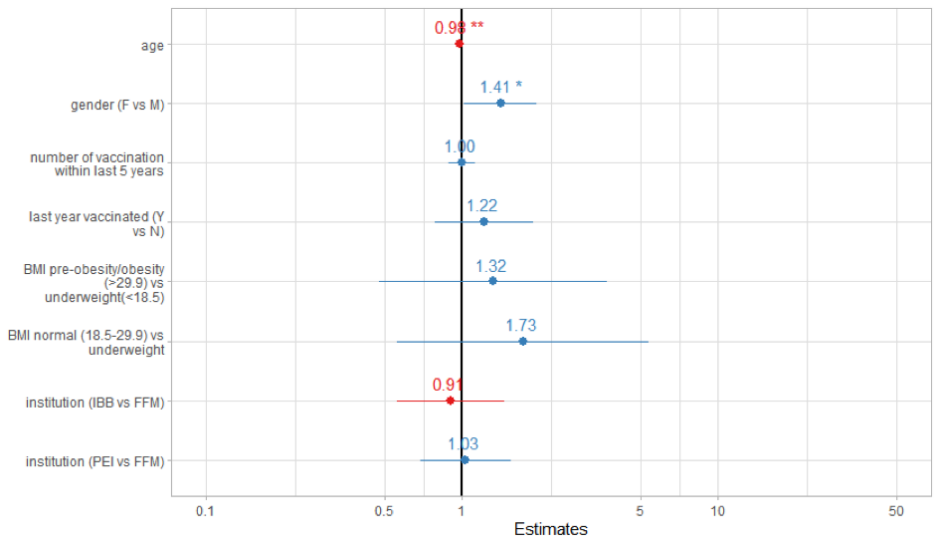
Factors associated with adverse event following immunization occurrence versus nonoccurrence. F: female; M; male; Y: yes; N: no; IBB: Investitionsbank Berlin; FFM: University Hospital Frankfurt; PEI: Paul Ehrlich Institute.

For the outcome reported AEFI occurrence, the results of the Cox regression indicated a negative association with increasing age (hazard ratio 0.98; CI 0.97-0.99) and a positive association with female individuals (hazard ratio 1.41; CI 1.01-1.96; Figure 6).

### Results of Additional Analysis

The hazard ratio for reporting an influenza-like illness at any time point within 3 months was 0.26 (CI 0.11-0.60) for persons who were vaccinated against influenza in the last year compared with those who were not vaccinated in the last year (Multimedia Appendix 1).

There were 6 users who responded to the usability questionnaire. The mean SUS score built out of 6 feedback questionnaire responders was 86.67 with no negative rating of the entertainment of the app, quality of information, or overall app suitability for reporting AEFIs.

## Discussion

### Principal Findings

Our study shows the feasibility of an app-based reporting of AEFIs and suggests an added value of a mobile app with near real-time features to report AEFIs for a period of 3 months. We found that adherence to app use was dependent on gender but independent of age and AEFI occurrence. On the other hand, determinants of a correct app entry were increasing age and higher education. The most observed AEFI types reported in our study were injection site pain, pain in extremity, and fatigue/asthenia.

### Comparison With Prior Work

In previous studies, several methods for active adverse events reporting have been tested, including diary cards and telephone interviews and a mobile app [15,23]. Compared with a study using diary cards and telephone interviews [23], the dropout rate in our study was lower. The attrition rate in our study is consistent with the one reported by Wilson et al [15], who also used a mobile app as a reporting system for reporting AEFIs. The high dropout rate in the study using telephone interviews and diary cards could be related to a lack of active reminders. In our research and in Wilson et al [15], reminder notifications were implemented in the apps. This is of relevance especially for newly introduced vaccines for which the reporting period immediately after vaccination is often a crucial interval. For a 1-week interval, for example, our app showed an attrition rate of less than 2%. Common determinants of attrition in longitudinal studies are sex and age, with males and younger age being more likely to discontinue their participation [24,25]. However, our results showed that female sex was associated with attrition, whereas age had no influence. In our project, participants adapted to a new technology in order to stay adherent in a longitudinal study. The known factors associated with the nonadaption of new technologies are female sex and older age [26,27]. Therefore, it is not surprising that our results showed different determinants for attrition than in common longitudinal studies.

Vaccine-related information, such as the batch number, is often incorrectly entered when done manually [28]. Accordingly, one-third of our study participants reported they struggled with entering those details into the SafeVac app. For future studies or routine recording, the use of a mobile phone camera as barcode scanner could be an alternative to capture such information on vaccinations [29-31].

Older age of vaccinees, child age, and female sex are known triggers for AEFIs [32]. These particular age groups were not included in our study. This could explain why we found a decrease in reported AEFIs with increasing age. We cannot rule out to what extent AEFI occurrence is due to actual occurrence or reporting behavior. However, this influences any kind of voluntary reporting.

In our study, we also asked participants to indicate previous influenza vaccinations. The uptake of the most recent previous vaccination was related to low reporting of an influenza-like illness as an AEFI within 3 months, whereas the overall number of influenza vaccination uptakes within the last 5 years seemed to have no effect.

The most observed types of AEFIs reported in our study (ie, injection site pain, pain in extremity, and fatigue/asthenia) correspond with other safety studies of influenza vaccinations [33]. One clinical trial, however, reported injection site pain, headaches, and myalgia more often (8% to 23% more often) than in our study [34]. The reasons for these differences are unclear. However, one possible reason could be that our study population might not be as healthy as the study population selected for the clinical trial.

When analyzing the kind of AEFI occurrence in relation to adherence level of app users/study participants (ie, fully and partially adherent users), we did not find a difference. Therefore, we conclude that no specific AEFI type was responsible for attrition. Furthermore, most of the attrition was toward the end of the study, leading to a similar likelihood of AEFI type occurrence in both groups.

### Strengths and Limitations

The main strengths of our feasibility study are its multicentric study nature, long observation time, and use of a custom designed app for reporting AEFIs. However, the study did not aim to provide generalizable results. In our study, we could demonstrate that individuals are willing to report AEFIs for more than the usually implemented follow-up period of 2 weeks to 1 month for active influenza AEFI reporting [14,15]. With the help of additional studies, this could mean that a broader spectrum of AEFIs (ie, unexpected late-onset AEFIs) could be captured with an app. In addition, by using a longitudinal approach, we were able to generate determinants of adherence to app use and information on AEFIs occurring after an annual influenza vaccination. With another study design (eg, use of app in routine vaccination settings and for different vaccines), a more diverse and increased participant group (eg, elderly) could be captured. This would help to assess the causality of AEFIs, their frequencies, seriousness, and course. A general limitation in our findings is the fact that we cannot disentangle how and which determinants influence the reporting and factual occurrence of AEFIs.

Given that the mean SUS score of all respondents was more than 85/100, the app’s usability seemed to be excellent according to the adjective scale rating [18]. However, we distributed the questionnaire to assess the usability and quality of the mobile app 1 year after recruitment due to difficulties in separating the feedback questionnaire from the national databank of PEI. The time delay between vaccination and questionnaire distribution could have led to a low response rate. In our study, we recruited participants face to face and used participation in a lottery as an incentive measure. Therefore, it is unclear if the same response and adherence rate can be expected outside of the study. Nevertheless, in order for participants to qualify for lottery participation, AEFI reports were required until 1 week after vaccination. As we do not see any immediate drop after that time point, we would exclude the incentive as the main reason behind the adherence to the app until the end of the study.

Additionally, our reported adverse event must be interpreted as such and not as an adverse reaction, meaning without any causality assessment. For that purpose, a comparison group with no vaccine would have been required.

### Conclusions

We have shown that the use of a mobile app to report AEFIs for 3 months was feasible for more than 60% of participants, and the most reported adverse event after influenza vaccination were similar to those reported in clinical trials. Future studies could use the SafeVac concept for enhanced AEFI reporting, especially by including broader target groups (eg, elderly people and children) and by implementing the app in routine settings for various vaccines (eg, in general practitioners’ offices). In addition, new vaccines like the one against COVID-19 can benefit from using an approach like SafeVac for safety reporting. In fact, SafeVac 2.0 was recently adapted to the COVID-19 vaccination and is used as the national reporting tool for AEFIs in an active surveillance study in Germany [35].

## Appendix

Multimedia Appendix 1 Factors associated with occurrence versus nonoccurrence of influenza-like illness.

## Abbreviations

AEFI: adverse event following immunization
OR: odds ratio
PEI: Paul Ehrlich Institute
SUS: System Usability Scale
