# Supplementary figures and images for: Postmarketing Safety Monitoring After Influenza Vaccination Using a Mobile Health App: Prospective Longitudinal Feasibility Study
Source: JMIR Mhealth Uhealth. 2021 May 7;9(5):e26289. doi: 10.2196/26289 (PMC8140379; doi:10.2196/26289)

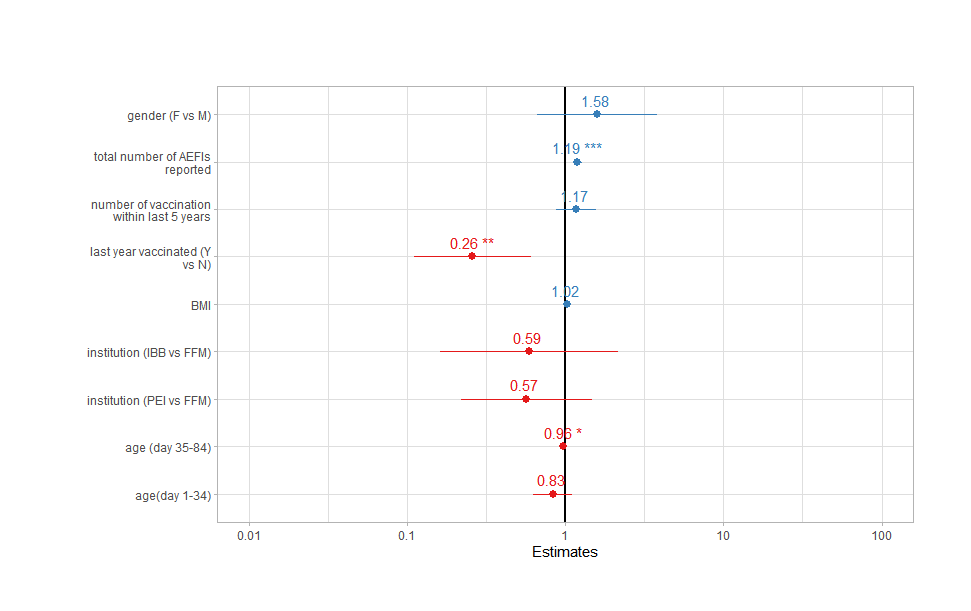

Supplement: Multimedia Appendix 1 [file mhealth_v9i5e26289_app1.png]
